# Immune–endothelial–coagulation crosstalk as a driver of multi-organ dysfunction in severe viral pneumonia

**DOI:** 10.3389/fimmu.2026.1878054

**Published:** 2026-08-21

**Authors:** Yuehui Jia, Yide Chen, Jiabao Liao, Fei Qu, Yanming Lu, Chunxiang Niu, Feng Chen

**Affiliations:** 1 Department of Emergency Medicine, Jiaxing Hospital of Traditional Chinese Medicine, Zhejiang, China; 2 Department of Pulmonary and Critical Care Medicine, First Affiliated Hospital of Kunming Medical University, Kunming, Yunnan, China; 3 Emergency Department, Jiaxing Hospital of Traditional Chinese Medicine, Jiaxing, China; 4 Yunnan University of Chinese Medicine, Yunnan, China

**Keywords:** coagulation dysregulation, endothelial injury, immunothrombosis, microcirculatory dysfunction, multi-organ dysfunction, viral pneumonia

## Abstract

Viral burden or pathogen identity alone cannot adequately explain the progression of severe viral pneumonia from a compartmentalized respiratory infection to acute respiratory distress syndrome, multi-organ failure, and death. Maladaptive immunity, endothelial damage, and coagulation dysregulation are all functionally integrated in a host-driven pathological mechanism that mediates disease escalation. Systemic microvascular damage and pulmonary inflammation are linked by immune-endothelial-coagulation interaction. This review investigates the ways in which immunothrombosis and microcirculatory dysfunction are propagated by defective antiviral immunity, alveolar–capillary barrier failure, damage-associated molecular pattern and neutrophil extracellular trap release, endothelial glycocalyx degradation, complement–platelet interactions, coagulation cascade activation, and impaired fibrinolysis. Lung-derived inflammatory signals cause endothelial activation and procoagulant reprogramming in distal organs following systemic dissemination, resulting in organ-specific phenotypes such as acute kidney injury, secondary myocardial injury, ARDS in the lung, neurovascular unit dysfunction, and barrier-disruption-associated inflammatory amplification along the liver–gut axis. This framework may provide a rationale for exploring stage-adapted and phenotype-guided approaches to severe viral pneumonia, including early antiviral therapy, immunomodulation during disease progression, endothelial–coagulation axis targeting, and host-directed strategies. Further longitudinal cohorts, multi-omics analyses, mechanism-based stratification studies, and mechanism-embedded clinical trials will be needed to determine whether immune–endothelial–coagulation coupling can be translated from a mechanistic model into a clinically actionable framework for precision intervention.

## 1 Introduction

Viral pneumonia remains a major cause of acute respiratory failure and infection-related mortality worldwide, with higher susceptibility reported in children, older adults, and immunocompromised individuals (1). Despite advances in antiviral therapy, respiratory support, and intensive care management, localized respiratory infection may progress to acute respiratory distress syndrome (ARDS), circulatory dysfunction, and multi-organ failure (2). Inter-patient heterogeneity after exposure to similar pathogens indicates that viral burden, anatomical site of infection, and pathogen identity do not sufficiently explain disease progression (3). Maladaptive immunity, endothelial damage, and coagulation dysregulation are all functionally integrated in severe illness, which has been described as a host-response disorder (4). Viral pneumonia’s pathogenic spectrum goes beyond decreased gas exchange, inflammatory exudation, and damage to the alveolar epithelium (5). Alveolar capillary barrier disruption, systemic inflammatory mediator dissemination, endothelial activation, aberrant microcirculatory perfusion, and coagulation–fibrinolysis imbalance have all been documented in severe cases (6). The lung is a source of systemic inflammatory signals as well as the location of viral replication and first immune activation (7). The distal organ endothelium and microvascular beds receive circulating cytokines, chemokines, damage-associated molecular patterns (DAMPs), and activated immune cells, which transform compartmentalized pulmonary infection into systemic immunovascular injury (8).

**Figure 1:**
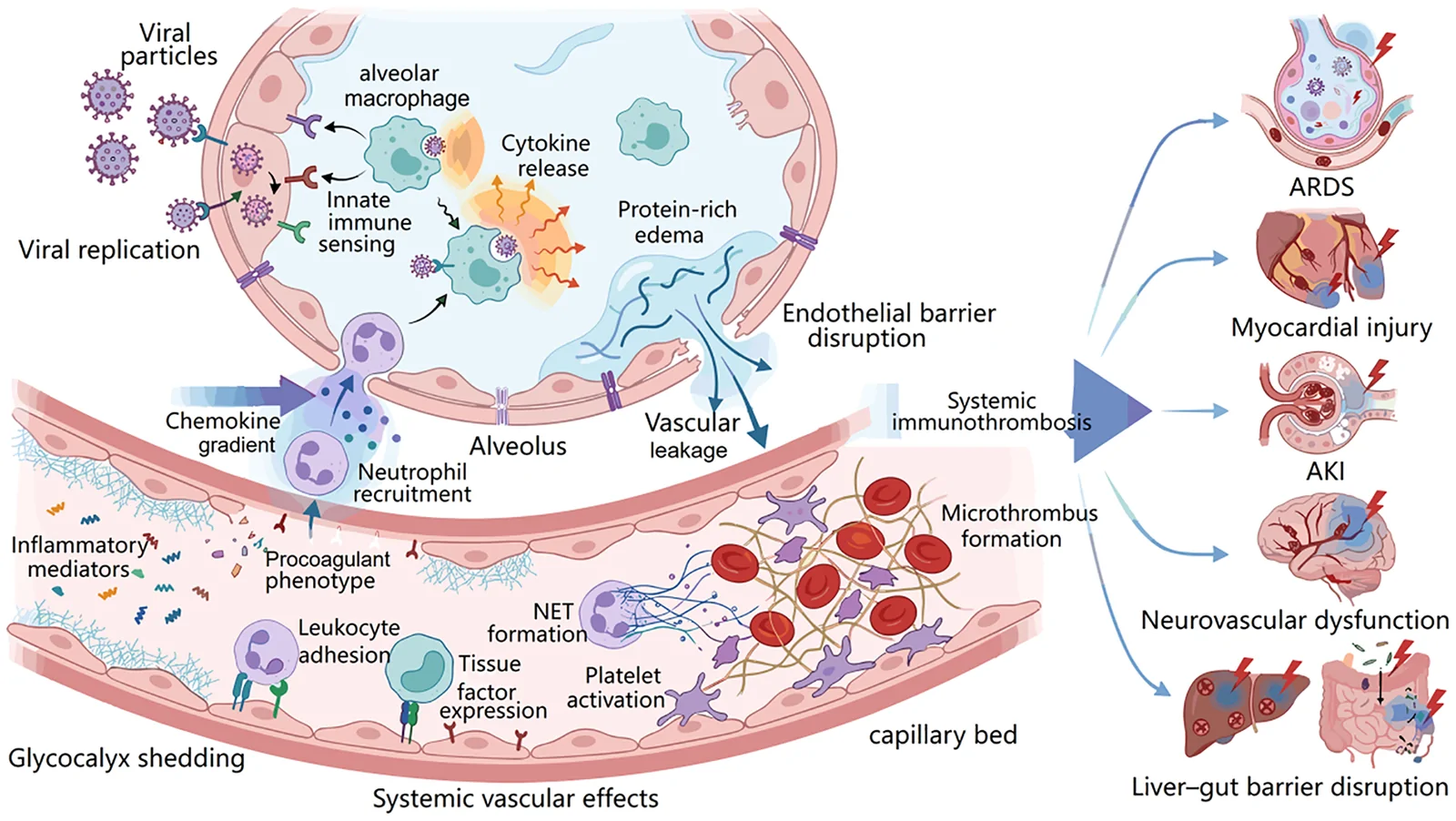
Immune–endothelial–coagulation crosstalk drives multi-organ injury in severe viral pneumonia. Respiratory viral infection initiates alveolar viral entry and replication, followed by activation of resident macrophages and innate immune sensing. Inadequate antiviral control promotes excessive cytokine production and neutrophil recruitment. Alveolar–capillary barrier failure increases vascular permeability and protein-rich edema, enabling inflammatory mediators to enter the circulation. These changes are accompanied by systemic endothelial activation, glycocalyx loss, leukocyte adhesion, and acquisition of a procoagulant phenotype. Endothelial injury, together with neutrophil extracellular trap (NET) formation and platelet activation, promotes fibrin deposition and microthrombus formation. The resulting immunothrombosis impairs microvascular perfusion and oxygen delivery, leading to tissue hypoxia and organ injury. Organ-specific manifestations include acute respiratory distress syndrome (ARDS) in the lung, myocardial injury in the heart, acute kidney injury (AKI), neurovascular dysfunction in the brain, and barrier disruption along the liver–gut axis.

One of the main mechanisms underpinning this change is immune-endothelial-coagulation crosstalk (9). Viral infection and inadequate antiviral control cause sustained innate immune activation; neutrophils, monocytes/macrophages, complement pathways, and inflammasomes all contribute to the amplification of the inflammatory cascade (10). Glycocalyx degradation, increased vascular permeability, decreased anticoagulant capacity, and increased expression of adhesion molecules are characteristics of endothelial damage, which is linked to inflammatory mediator release, oxidative stress, and complement activation (11). Endothelial dysfunction increases platelet activation, tissue factor expression, and the start of the coagulation cascade (12). NETs connect innate immune activation to immunothrombosis by acting as a structural scaffold for platelet adhesion and fibrin deposition. Microthrombus deposition, perfusion heterogeneity, and tissue hypoxia are effector processes that mediate multi-organ failure during the systemic spread of this host-defense response (8, 13) (**Figure 1**).

A pathogenic continuum comprising lung-derived inflammatory signaling, endothelial damage, immunothrombosis, microcirculatory dysfunction, and multi-organ failure can be used to describe severe viral pneumonia (14). Myocardial damage, acute kidney injury, neuroinflammation, liver-gut barrier failure, and coagulation abnormalities are examples of extrapulmonary symptoms that are organ-specific results of a common immune-vascular-coagulation network among different tissue microenvironments (15). The variety of organ phenotypes may be influenced by variations in immune-cell composition, metabolic requirement, microvascular organization, and barrier architecture. Key mechanistic gaps remain, including the checkpoints through which lung-derived inflammatory signals breach local barriers and induce distal endothelial dysfunction, the relative contribution of immunothrombosis to individual organ injury, and the stage-specific alignment of antiviral therapy, immunomodulation, anticoagulation, and endothelial-protective interventions (16). Biomarkers related to inflammation, coagulation, and endothelial injury have been proposed, whereas their longitudinal validation and mechanism-based stratification capacity remain limited. In this Review, immune–endothelial–coagulation crosstalk is used as the central framework to synthesize the mechanisms by which viral pneumonia progresses from localized infection to systemic immunothrombosis and multi-organ dysfunction. Interactions among defective antiviral immunity, alveolar–capillary barrier failure, endothelial injury, NET–platelet–complement signaling, coagulation–fibrinolysis imbalance, and microcirculatory dysfunction are examined, with additional discussion of organ-specific manifestations and stratified therapeutic strategies. This framework may support mechanism-based classification and host-directed therapeutic optimization in severe viral pneumonia (8, 17). Importantly, immune–endothelial–coagulation coupling is not presented here as a newly discovered biological pathway. Its individual components—including dysregulated antiviral immunity, endothelial activation and injury, complement and platelet activation, NET formation, coagulation–fibrinolysis imbalance, and immunothrombosis—have been described in previous mechanistic, pathological, and clinical studies (18). The conceptual contribution of this Review is to integrate these previously studied processes into a unified and dynamic pathogenic continuum linking lung-derived inflammatory spillover to endothelial reprogramming, immunothrombosis, microcirculatory dysfunction, and organ-specific injury (19). By further connecting this mechanistic continuum with disease stage, dominant pathological phenotype, and therapeutic windows, the framework is intended to provide an organizational model for understanding how localized viral pneumonia progresses toward systemic multi-organ dysfunction. Thus, the contribution of the framework lies primarily in the integration, temporal organization, and cross-organ interpretation of existing evidence rather than in proposing that its individual biological components are newly identified mechanisms.

### 1.1 Literature search and review strategy

This narrative review was based on literature identified through searches of PubMed, Web of Science, and Google Scholar, primarily covering publications from the preceding 10 years (approximately 2016–2026, up to the final search date). The literature search used combinations of terms related to severe viral pneumonia, acute respiratory distress syndrome, antiviral immune responses, endothelial injury and dysfunction, coagulation dysregulation, immunothrombosis, neutrophil extracellular traps, complement activation, microcirculatory dysfunction, and multi-organ injury. Studies were selected primarily according to their relevance to the immune–endothelial–coagulation framework developed in this review. Mechanistic studies, clinical and pathological investigations, translational studies, and relevant reviews were considered when they provided evidence regarding the initiation, amplification, systemic dissemination, organ-specific consequences, or therapeutic implications of this coupled network. Because this article was designed as a narrative rather than a systematic review, literature selection and synthesis were guided by mechanistic relevance and contribution to the conceptual framework, without application of a formal systematic-review or meta-analytic selection protocol.

## 2 Lung-derived inflammatory spillover

The progression of viral pneumonia from compartmentalized respiratory infection to systemic pathology has been associated with a pulmonary amplification network defined by defective antiviral control, epithelial–endothelial barrier disruption, and injury-signal release (20). The lung is situated where coagulation, vascular control, and immune activation come together. The alveolar microenvironment releases cytokines, damage-associated molecular patterns (DAMPs), activated immune cells, and coagulation-associated mediators into the systemic circulation when antiviral control is compromised or inflammation is prolonged (6). These lung-derived signals are delivered to distal vascular endothelium and microcirculatory beds, where systemic immune–endothelial–coagulation crosstalk is initiated. Lung-derived inflammatory spillover is implicated as an early event in multi-organ injury and in the transition from local pulmonary inflammation to systemic microvascular pathology.

Viral pneumonias vary in cellular tropism, replication kinetics, immune-evasion strategies, and susceptible host populations, with corresponding differences in pulmonary pathology and extrapulmonary involvement. Severe viral pneumonia has been characterized by dysregulated interferon responses, epithelial–endothelial barrier failure, inflammatory myeloid-cell recruitment, complement and coagulation activation, NET formation, and microvascular injury in clinical and pathological studies. The major types of viral pneumonia, together with their immunopathological features and extrapulmonary manifestations, are summarized in **Table 1**. The shared pathological processes underlying progression from viral pneumonia to multi-organ injury are therefore examined across three mechanistic layers: dysregulated antiviral responses, epithelial–endothelial barrier disruption, and DAMP/NET release coupled to local immunothrombosis, rather than by individual viral pathogen. Although these processes provide a shared conceptual framework for severe viral pneumonia, the relative contribution of each component is pathogen dependent, and the supporting evidence is not equally developed across viral infections. SARS-CoV-2 infection has provided the most extensive clinical and pathological evidence for endothelial activation, complement–platelet interactions, NET-associated immunothrombosis, coagulation–fibrinolysis imbalance, and systemic microvascular injury (21). Influenza pneumonia shares several upstream features, including epithelial injury, dysregulated interferon responses, neutrophil and monocyte recruitment, inflammatory cytokine release, and coagulation activation; however, secondary bacterial infection and extensive epithelial damage may contribute more prominently to disease progression (22). In RSV and HMPV infection, disease is more strongly characterized by epithelial innate immune activation, mucus-associated inflammation, small-airway obstruction, and Th2/Th17-related responses, whereas direct evidence for systemic NET-driven immunothrombosis and endothelial–coagulation coupling is comparatively limited (23). Severe adenovirus pneumonia is frequently associated with epithelial-cell necrosis, sustained cytokine release, lymphocyte dysfunction, and DAMP release, which may secondarily promote vascular inflammation rather than reproduce the full immunothrombotic phenotype characterized in COVID-19 (24). Other viral pneumonias may show distinct vascular patterns; for example, highly pathogenic or emerging viruses can produce prominent endothelial barrier disruption, capillary leakage, hypoperfusion, and coagulation abnormalities (25). Thus, immune–endothelial–coagulation crosstalk should be regarded as a shared pathophysiological framework whose dominant nodes and relative intensity vary according to viral tropism, host immune responses, disease stage, and host susceptibility, rather than as an identical mechanistic sequence across all viral pneumonias.

**Table 1:** Major viral pneumonia types, immunopathological features, and differential routes to extrapulmonary organ injury.

Viral category	Representative pathogens	Pulmonary pathological phenotype	Immunopathological features	Extrapulmonary involvement	Dominant coupling node (s) and characteristic route to extrapulmonary injury
Coronavirus-associated pneumonia	SARS-CoV-2, SARS-CoV, MERS-CoV	Diffuse alveolar damage, ARDS, alveolar–capillary barrier disruption, and pulmonary microvascular injury (26)	Dysregulated IFN responses, activation of inflammatory monocytes/macrophages, complement involvement, endothelial activation, and coagulation abnormalities (27, 28)	Myocardial injury, AKI, liver injury, neurological involvement, and gastrointestinal involvement (29)	Endothelial–coagulation and thromboinflammatory mechanisms are particularly prominent, especially in severe SARS-CoV-2 infection. Pulmonary inflammatory spillover may promote systemic endothelial activation, complement–platelet signaling, NET-associated immunothrombosis, and coagulation–fibrinolysis imbalance. These processes, together with hypoxemia and systemic inflammation, provide a route to diffuse microvascular injury involving the heart, kidney, liver, nervous system, and gastrointestinal tract.
Influenza virus pneumonia	H1N1, H5N1, H7N9, and other influenza viruses	Necrotizing bronchitis, viral pneumonia, diffuse alveolar damage, and ARDS (30)	Epithelial injury, dysregulated IFN responses, chemokine release, neutrophil and monocyte recruitment, and secondary infection (31)	Myocardial injury, AKI, liver injury, encephalopathy, or neuroinflammation (32)	Epithelial injury and inflammatory amplification appear to have greater relative weight. Extensive pulmonary epithelial damage, dysregulated antiviral responses, inflammatory-cell recruitment, severe hypoxemia, and, in some patients, secondary bacterial infection may drive systemic deterioration. Coagulation activation can contribute, but extrapulmonary organ injury may arise from the combined effects of severe pulmonary injury, systemic inflammation, infection-related complications, hypoxemia, and hemodynamic stress rather than from a uniformly dominant immunothrombotic phenotype.
RSV/HMPV-associated pneumonia	RSV, HMPV	Bronchiolitis, mucus plugging, small-airway obstruction, and hypoxic lung injury (33)	Activation of epithelial innate immunity, mucus-associated inflammation, Th2/Th17-related responses, and inflammatory-cell infiltration (34)	Severe lower respiratory tract infection, hypoxic respiratory failure, cardiopulmonary deterioration, or secondary systemic inflammatory responses in infants, older adults, and immunocompromised individuals (35, 36)	Airway epithelial inflammation, small-airway obstruction, and hypoxia are the predominant features. Extrapulmonary deterioration may therefore occur mainly through respiratory failure, hypoxic cardiopulmonary stress, and extension of local inflammation into the systemic compartment. Compared with severe SARS-CoV-2 infection, direct evidence supporting widespread NET-driven immunothrombosis and systemic endothelial–coagulation injury is comparatively limited.
Adenovirus pneumonia	HAdV-3, HAdV-7, HAdV-55, and other adenoviruses	Necrotizing pneumonia, pulmonary consolidation, diffuse alveolar damage, and ARDS (37)	Epithelial-cell necrosis, sustained cytokine release, lymphopenia or impaired lymphocyte function, and release of tissue-injury signals (24)	Liver injury, myocardial injury, encephalitis, and gastrointestinal involvement (38, 39)	Tissue necrosis, DAMP release, and sustained inflammatory signaling represent prominent upstream mechanisms. Extensive epithelial and tissue injury may generate DAMP-mediated inflammatory amplification, while lymphocyte dysfunction may impair viral control. Hepatic, myocardial, neurological, and gastrointestinal injury may consequently reflect the combined effects of tissue necrosis, systemic cytokine signaling, and secondary vascular inflammation rather than a predominantly NET-driven immunothrombotic process.
Herpesvirus-associated pneumonia	CMV, HSV, VZV	Interstitial pneumonia, necrotizing pneumonia, and diffuse lung injury (40, 41)	Viral reactivation, impaired T-cell immunity, monocyte–macrophage inflammatory responses, and endothelial injury (42)	Hepatitis, encephalitis, gastrointestinal injury, and bone-marrow suppression (43)	Impaired cellular immune control and viral reactivation are major upstream determinants, particularly in immunocompromised hosts. Extrapulmonary injury may reflect a combination of virus-associated tissue involvement, persistent monocyte–macrophage inflammation, and endothelial injury. This pattern may produce hepatic, neurological, gastrointestinal, and hematological manifestations that differ from the predominantly microvascular thromboinflammatory pattern described in severe COVID-19.
Highly pathogenic or emerging viral pneumonia	Hantavirus, Nipah virus, and other emerging viruses	Pulmonary edema, ARDS, capillary leakage, and hemorrhagic lung injury (44)	Endothelial-cell activation or injury, increased vascular permeability, cytokine release, and coagulation abnormalities (45)	Kidney injury, neurological injury, shock, and circulatory failure (46, 47)	Endothelial barrier failure, vascular leakage, and hypoperfusion may dominate systemic progression. Increased vascular permeability and capillary leakage can promote intravascular volume loss, tissue edema, impaired organ perfusion, coagulation abnormalities, and shock. Consequently, renal injury, neurological dysfunction, and circulatory failure may be driven more strongly by vascular barrier disruption and perfusion failure than by the same immunothrombotic sequence emphasized in severe SARS-CoV-2 infection.

ARDS, acute respiratory distress syndrome; AKI, acute kidney injury; IFN, interferon; RSV, respiratory syncytial virus; HMPV, human metapneumovirus; CMV, cytomegalovirus; HSV, herpes simplex virus; VZV, varicella-zoster virus; DAMPs, damage-associated molecular patterns; NETs, neutrophil extracellular traps. The pathogens and extrapulmonary manifestations listed in the table are representative examples and do not provide an exhaustive summary of all viral pneumonia types or clinical presentations. The relative mechanistic weighting shown in the final column is intended to highlight pathogen-associated differences within the shared immune–endothelial–coagulation framework; it should not be interpreted as indicating that these pathways are mutually exclusive or equally established across all viral infections.

### 2.1 Pathogen-specific weighting of the coupled network and differential multi-organ injury

Although severe viral pneumonias may converge on a shared immune–endothelial–coagulation network, different viruses enter and amplify this network through distinct dominant mechanisms, which may in turn shape the pattern of extrapulmonary organ injury. The resulting multi-organ phenotypes should therefore be regarded as convergent clinical endpoints generated by pathogen-dependent weighting of epithelial injury, immune activation, endothelial dysfunction, coagulation abnormalities, hypoxemia, and systemic inflammatory dissemination rather than as manifestations of an identical pathogenic sequence. In coronavirus-associated pneumonia, particularly SARS-CoV-2 infection, endothelial activation, complement–platelet interactions, coagulation–fibrinolysis imbalance, and NET-associated immunothrombosis are prominent features of severe disease (48). This vascular and thromboinflammatory component provides a mechanistic link to the broad extrapulmonary phenotype involving the heart, kidney, liver, nervous system, and gastrointestinal tract. In this setting, systemic endothelial dysfunction and microvascular thrombotic injury may act alongside hypoxemia and inflammatory signaling to distribute injury across multiple vascular beds (29). Influenza pneumonia appears to place relatively greater weight on extensive respiratory epithelial injury, dysregulated interferon responses, neutrophil and monocyte recruitment, cytokine release, and, in some patients, secondary bacterial infection (22). Coagulation activation can accompany severe influenza, but systemic organ dysfunction may arise through a broader combination of severe pulmonary injury, hypoxemia, systemic inflammation, secondary infection, and hemodynamic stress. These mechanisms may contribute to myocardial injury, AKI, hepatic dysfunction, encephalopathy, and neuroinflammatory manifestations without necessarily reproducing the endothelial–immunothrombotic dominance observed in severe COVID-19 (32). RSV- and HMPV-associated disease is weighted more strongly toward epithelial innate immune activation, mucus-associated inflammation, small-airway obstruction, and Th2/Th17-related responses (49). Consequently, extrapulmonary deterioration in severe cases may be driven predominantly by respiratory failure, hypoxic stress, cardiopulmonary decompensation, and extension of local inflammation into the systemic compartment, particularly in infants, older adults, and immunocompromised individuals. Compared with SARS-CoV-2, evidence supporting a major role for systemic NET-driven immunothrombosis or widespread endothelial–coagulation injury remains more limited. Adenovirus pneumonia represents a somewhat different pattern in which extensive epithelial-cell necrosis, sustained cytokine release, lymphocyte dysfunction, and DAMP liberation can produce marked tissue-inflammatory injury. Liver injury, myocardial injury, encephalitic manifestations, and gastrointestinal involvement may therefore reflect a combination of tissue necrosis, systemic inflammatory signaling, and secondary vascular inflammation, rather than a predominantly thromboinflammatory mechanism (50). In herpesvirus-associated pneumonia, particularly in immunocompromised hosts, viral reactivation and impaired T-cell control are prominent upstream features; hepatitis, encephalitis, gastrointestinal injury, and bone-marrow suppression may accompany pulmonary disease, with monocyte–macrophage inflammation and endothelial involvement contributing to systemic injury (51). By contrast, some highly pathogenic or emerging viral infections may preferentially engage the vascular component of the network through endothelial barrier disruption and increased vascular permeability. In these settings, capillary leakage, coagulation abnormalities, shock, and impaired organ perfusion may become dominant determinants of kidney injury, neurological dysfunction, and circulatory failure (25). Thus, the relative position of each virus within the immune–endothelial–coagulation framework differs: some infections are weighted toward epithelial and inflammatory injury, others toward endothelial–coagulation dysfunction, and others toward profound barrier failure or virus-specific tissue injury. These pathogen-dependent differences help explain why severe viral pneumonias can share a common systemic framework while producing distinct patterns and relative frequencies of multi-organ dysfunction.

### 2.2 Dysregulated antiviral responses and inflammatory amplification

Following respiratory viral entry, viral nucleic acids and associated molecular patterns are detected by host pattern recognition receptors (PRRs), triggering interferon (IFN)-mediated antiviral programs. Transcriptional regulators like NF-κB and IRF3/7 cause type I and type III IFNs, interferon-stimulated genes (ISGs), and antiviral effector molecules when nucleic acid-sensing systems, such as Toll-like receptors (TLRs), RIG-I-like receptors (RLRs), and the cGAS–STING axis, are activated (52). Controlling viral replication, limiting infectious dissemination, and maintaining local inflammatory homeostasis all depend on an early, proportional, and temporally coordinated IFN–ISG response (10).

Temporal dysregulation of antiviral immunity is often seen in severe viral pneumonia. Delayed or inadequate early IFN responses impair viral clearance and permit persistent viral replication, epithelial injury, and inflammatory mediator release. During the middle and late phases of infection, sustained activation of IFN- and NF-κB-associated inflammatory signaling can convert antiviral defense into tissue injury and immunopathological amplification (53). Early antiviral barriers are weakened by respiratory viruses through suppression of IFN production, disruption of JAK–STAT signaling, impaired antigen presentation, and evasion of immune recognition, thereby establishing a positive-feedback process defined by insufficient viral clearance, persistent inflammatory activation, and progressive tissue injury (54). In parallel, pulmonary innate immune-cell recruitment and activation are increased, with enhanced release of cytokines and chemokines, including IL-6, TNF-α, CXCL8, and CCL2, which promote the accumulation of neutrophils, monocytes, and macrophages at infected sites (55). Increased IL-6, C-reactive protein, ferritin, lactate dehydrogenase, and neutrophil-to-lymphocyte ratio have been associated with disease severity, oxygenation impairment, and adverse outcomes (56).

Inflammasome signaling constitutes an additional layer of local inflammatory amplification. The NLRP3 inflammasome is triggered by viruses, ionic dyshomeostasis, mitochondrial damage, and cell-damage signals. This leads to the activation of caspase-1, the maturation of IL-1β and IL-18, and gasdermin D-mediated pyroptosis (57). Pulmonary antiviral immunity becomes a self-sustaining inflammatory network when pyroptotic cells generate DAMPs and inflammatory mediators that activate nearby immune cells. Dysregulated antiviral responses therefore provide an immunological substrate for endothelial activation, coagulation initiation, and lung-derived inflammatory spillover, while also determining the extent of local lung injury.

### 2.3 Epithelial–endothelial barrier disruption and inflammatory spillover

The alveolar epithelium and capillary endothelium form the alveolar–capillary barrier, a specialized interface required for gas exchange, fluid homeostasis, and local immune compartmentalization (58). Barrier integrity is essential for spatial containment of pulmonary inflammation. During viral infection, alveolar epithelial injury is accompanied by apoptosis, necrosis, pyroptosis, and functional impairment, together with reduced expression of tight-junction proteins, including ZO-1, occludin, and claudin-18, resulting in impaired epithelial barrier stability (59). Alveolar protein leakage, defective alveolar fluid clearance, and decreased PaO_2_/FiO_2_ ratios have been associated with barrier injury and ARDS severity (60).

The pulmonary microvascular endothelium serves as a major interface for the systemic dissemination of lung-derived inflammatory signals. Pulmonary endothelial activation is induced by inflammatory cytokines, complement fragments, DAMPs, and local hemodynamic alterations, and is characterized by increased ICAM-1, VCAM-1, and E-selectin expression, enhanced leukocyte adhesion, increased vascular permeability, and conversion from an anticoagulant to a procoagulant phenotype (61). Increased syndecan-1, angiopoietin-2, von Willebrand factor, soluble thrombomodulin, and D-dimer indicate endothelial glycocalyx degradation, endothelial activation, endothelial injury, and coagulation–fibrinolysis imbalance (62). Endothelial activation thereby links immune amplification, coagulation initiation, and microcirculatory dysfunction.

After epithelial–endothelial barrier disruption, the spatial containment of pulmonary inflammation is lost. Locally generated inflammatory mediators and DAMPs, including IL-6, TNF-α, IL-1β, HMGB1, mitochondrial DNA, and S100A8/A9, are disseminated through the circulation and delivered to distal organ endothelium and microvascular beds, where systemic endothelial activation and secondary immune–coagulation responses are initiated (63). This process has been associated with increased SOFA scores, greater ARDS severity, acute kidney injury, and multi-organ dysfunction (64). Barrier disruption therefore constitutes both a pathological basis for hypoxemia and ARDS and a structural prerequisite for lung-derived inflammatory spillover and systemic microvascular injury.

### 2.4 DAMP/NET release and local immunothrombosis

DAMP and NET release has been implicated in the transition from inflammatory amplification to immunothrombosis in viral pneumonia (26). Viral infection, hypoxia, oxidative stress, and inflammatory stimulation induce necrosis, pyroptosis, and mitochondrial dysfunction in alveolar epithelial cells, endothelial cells, and immune cells, with subsequent release of damage-associated molecular patterns (DAMPs), including mitochondrial DNA (mtDNA), ATP, HMGB1, and oxidatively modified molecules (65). Increased mtDNA and HMGB1 have been used as markers of cell-damage-associated signaling and have been linked to inflammatory intensity and disease severity (66). Extracellular mtDNA is sensed by cGAS and activates STING signaling, leading to type I IFN induction and inflammatory mediator production; ATP and HMGB1 engage pattern recognition receptors and promote inflammasome activation (67). Sustained DAMP release maintains local inflammatory signaling and is coupled to cell injury and secondary inflammatory amplification.

**Figure 2:**
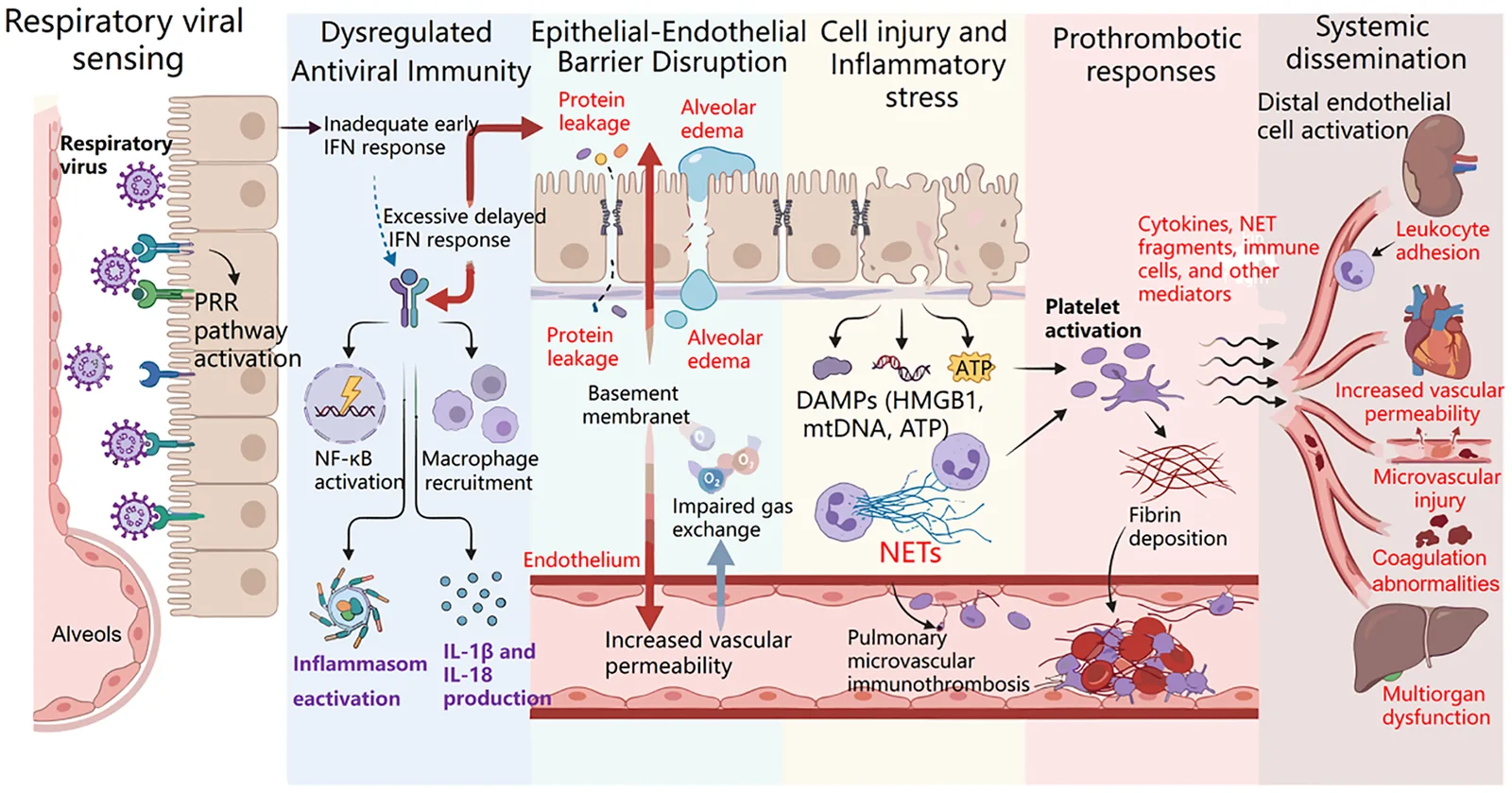
Lung-derived inflammatory spillover links defective antiviral control to systemic immune–vascular injury. Respiratory viral sensing activates pattern-recognition receptor (PRR) pathways in the airway and alveolar epithelium. Dysregulated antiviral immunity, characterized by inadequate early interferon (IFN) responses or excessive delayed IFN signaling, promotes NF-κB activation, macrophage recruitment, inflammasome activation, and IL-1β/IL-18 production. Epithelial–endothelial barrier disruption reduces junctional integrity, increases vascular permeability, causes protein leakage and edema, and impairs gas exchange. Cell injury and inflammatory stress induce damage-associated molecular pattern (DAMP) release, including HMGB1, mitochondrial DNA, and ATP, together with neutrophil extracellular trap (NET) formation. DAMPs and NETs promote platelet activation, fibrin deposition, and pulmonary microvascular immunothrombosis. Cytokines, DAMPs, NET fragments, immune cells, and soluble mediators then enter the systemic circulation, inducing distal endothelial activation, leukocyte adhesion, permeability changes, microvascular injury, coagulation abnormalities, and multi-organ dysfunction.

NET-associated components contribute to epithelial and endothelial injury, while the extracellular DNA scaffold facilitates platelet adhesion, coagulation-factor accumulation, and fibrin deposition, thereby linking inflammatory amplification to local pulmonary immunothrombosis (68). When inflammatory signaling persists, this initially protective response may contribute to heterogeneous capillary perfusion and ventilation–perfusion mismatch (69, 70). Thus, the DAMP–NET axis provides an early mechanistic bridge between pulmonary tissue injury, local immunothrombosis, and subsequent systemic immune–vascular signaling (**Figure 2**).

## 3 Endothelial injury

The vascular endothelium serves as a source of inflammatory signal amplification and pathological translation after lung-derived inflammatory signals enter the bloodstream (69). Circulating cytokines, complement fragments, damage-associated molecular patterns (DAMPs), and activated immune cells are delivered to endothelial cells, where structural and functional alterations are induced. Endothelial dysfunction is characterized by reduced barrier stability, increased permeability, attenuation of anticoagulant and anti-inflammatory phenotypes, and acquisition of pro-inflammatory and procoagulant features (71). The endothelial conversion of inflammatory cues is associated with systemic vasculopathy and microcirculatory imbalance, thereby establishing permissive conditions for immunothrombosis.

### 3.1 Inflammatory cytokines, complement, and endothelial activation

After lung-derived inflammatory signals enter the circulation, increased IL-6, TNF-α, CXCL8, and related cytokines are delivered to the vascular endothelium, where endothelial cells are shifted from a quiescent to an activated state (72). Endothelial activation is characterized by increased ICAM-1, VCAM-1, and E-selectin expression, enhanced leukocyte adhesion and transmigration, and induction of inflammation-related gene programs, which together promote immune-cell retention within the vascular wall and local inflammatory amplification (73). Angiopoietin-2 (Ang-2) provides a circulating marker of endothelial activation and vascular hyperpermeability, with higher levels associated with greater ARDS severity and organ dysfunction (74).

Complement signaling provides an additional amplification layer for endothelial inflammation. Complement fragments, particularly C3a and C5a, enhance endothelial inflammatory signaling, neutrophil recruitment, and vascular permeability (75). In parallel, circulating DAMPs such as mtDNA and HMGB1 activate endothelial pattern-recognition pathways and NF-κB signaling (69). Together, these inflammatory inputs shift endothelial cells from a quiescent barrier-maintaining state toward a pro-inflammatory phenotype characterized by enhanced leukocyte adhesion and impaired vascular homeostasis. The structural consequences of endothelial glycocalyx disruption and their effects on microcirculatory function are discussed in the following section.

### 3.2 Glycocalyx degradation, barrier disruption, and microcirculatory imbalance

Endothelial glycocalyx injury is linked to endothelial activation. The glycocalyx is a polysaccharide–protein complex covering the endothelial surface and is required for vascular permeability control, maintenance of anticoagulant properties, and shear-stress sensing (76). Glycocalyx degradation is induced by inflammatory mediators, oxidative stress, and protease release, resulting in loss of the anti-adhesive properties of the endothelial surface and increased leukocyte and platelet adhesion (77). Shedding of syndecan-1, heparan sulfate, and hyaluronan accompanies glycocalyx injury and has been linked to impaired vascular barrier function (78). Glycocalyx disruption and tight-junction dysfunction increase vascular permeability and are accompanied by plasma protein extravasation, interstitial edema, and inflammatory-cell infiltration (79).

Loss of capillary endothelial structural integrity is associated with altered perfusion patterns, including heterogeneous blood-flow distribution and local stasis. VE-cadherin junction disruption is linked to reduced endothelial barrier stability, tissue edema, and impaired oxygenation (80). Microcirculatory imbalance is shaped by inflammation–endothelium–coagulation interactions. Impaired endothelial barrier function permits increased tissue entry of inflammatory mediators and facilitates local accumulation of coagulation factors and platelets (81). From a clinical perspective, lactate provides an index of tissue hypoperfusion, whereas abnormalities in D-dimer, von Willebrand factor (VWF), and soluble thrombomodulin (sTM) capture complementary aspects of coagulation activation and endothelial injury; collectively, these alterations have been associated with organ dysfunction (74).

### 3.3 Loss of the anticoagulant phenotype and formation of procoagulant endothelium

Endothelial dysfunction is coupled to coagulation activation. After endothelial injury, anticoagulant properties are lost, whereas procoagulant pathways are induced (43). Upregulated tissue factor (TF) expression activates the extrinsic coagulation pathway and supports thrombin generation and fibrin deposition (82). Endothelial procoagulant conversion is further reflected by enhanced VWF release, which favors platelet adhesion and aggregation, while increased circulating soluble thrombomodulin (sTM) is consistent with disruption of endothelial anticoagulant function (70, 83).

Fibrinolytic suppression provides an additional mechanism for thrombus persistence. PAI-1 limits fibrin breakdown by inhibiting fibrinolytic activity, whereas D-dimer reflects ongoing fibrin turnover and the accompanying imbalance between coagulation and fibrinolysis (84, 85). Inflammation and coagulation are mutually reinforcing. While inflammatory mediators encourage the activation of the coagulation pathway, creating an inflammation–coagulation feed-forward loop, thrombin, fibrin, and platelets activate immune cells and enhance inflammatory signaling (16). Immunothrombosis, microvascular blockage, and decreased tissue perfusion are all consequences of these processes. Organ dysfunction is linked to microthrombus production and coagulation phenotypic change. Immunothrombi are stabilized by coagulation factor activation, platelet aggregation, and fibrinolytic inhibition, which are associated with multiple organ damage and poor pulmonary gas exchange (86).

## 4 Immunothrombosis

Following endothelial activation and procoagulant reprogramming, immunothrombosis represents a major effector phase of the immune–endothelial–coagulation continuum. At this stage, previously activated immune, endothelial, platelet, complement, and coagulation pathways converge within the microvasculature, shifting a locally protective host-defense response toward pathological thrombus persistence, perfusion failure, and tissue injury (87). The following sections therefore focus on the reciprocal amplification of NET–platelet–complement signaling, impaired anticoagulant and fibrinolytic regulation, and the downstream microcirculatory consequences of immunothrombosis.

### 4.1 NET–platelet–complement interactions

Within the pulmonary microvasculature, NETs interact reciprocally with activated platelets and complement to amplify immunothrombosis. Activated platelets promote further NET formation through P-selectin, CD40L, and pro-inflammatory signaling, whereas C3a and C5a enhance neutrophil activation, platelet aggregation, and endothelial inflammatory responses (88, 89). The resulting NET–platelet–complement feed-forward circuit promotes coagulation-factor accumulation, fibrin deposition, and thrombus stabilization, thereby converting upstream inflammatory activation into sustained microvascular obstruction. Persistent activation of this circuit contributes to heterogeneous pulmonary capillary perfusion, ventilation–perfusion mismatch, and impaired oxygenation (90). With systemic dissemination of inflammatory and coagulation signals, analogous interactions may also contribute to distal-organ microvascular injury.

### 4.2 Impaired fibrinolysis, coagulation cascade activation, and tissue factor

Coagulation activation occurs in tandem with inflammation in the alveolar-capillary unit during the development of severe viral pneumonia. Injured pulmonary microvascular endothelium, activated monocytes, and exposed subendothelial structures serve as sources of tissue factor (TF) (91). Increased TF expression initiates the extrinsic coagulation pathway, enhances thrombin generation and fibrin deposition, and increases the risk of pulmonary microvascular immunothrombosis (92).

Disrupted anticoagulant and fibrinolytic regulation promotes thrombus persistence. Diminished protein C pathway activity and reduced expression of anticoagulant molecules, together with increased circulating soluble thrombomodulin (sTM), are consistent with impaired anticoagulant function of the pulmonary microvascular endothelium (85). In parallel, PAI-1-mediated suppression of fibrinolysis restricts fibrin clearance, whereas D-dimer provides a measure of ongoing fibrin turnover and coagulation–fibrinolysis imbalance in severe viral pneumonia (93). Under a sustained procoagulant milieu, endothelial exposure of procoagulant phospholipids, platelet–NET complex formation, and complement-mediated inflammatory amplification promote pulmonary microthrombus stabilization. Fibrin deposition and platelet aggregation within the pulmonary microvasculature are associated with heterogeneous capillary perfusion, ventilation–perfusion mismatch, and aggravated hypoxemia (94). After systemic dissemination of inflammatory–coagulation signals, analogous processes may extend to distal organ microvascular beds and contribute to multi-organ dysfunction.

### 4.3 Microthrombus deposition, hypoperfusion, and organ energy crisis

Immunothrombosis is linked to microvascular perfusion failure. Microthrombi are deposited in capillary beds of the lung, heart, kidney, brain, and liver, where continuous flow is replaced by intermittent perfusion or vascular obstruction, leading to insufficient oxygen delivery and restricted metabolic substrate availability (95). During this process, VWF and D-dimer provide complementary evidence of microvascular thrombotic activity and systemic coagulation activation (96). In contrast to macrovascular thrombosis, microvascular perfusion defects are diffusely distributed, may occur without overt large-vessel thrombotic events, and are associated with widespread tissue injury (97).

Sustained hypoperfusion and inflammatory stress impose a metabolic burden on affected tissues. Glycolysis may replace oxidative phosphorylation in cellular metabolism, resulting in a decreased ability to produce ATP (98). Further limiting energy production, mitochondrial malfunction increases the creation of reactive oxygen species and causes cellular damage (99). Rising lactate may reflect the combined effects of tissue hypoperfusion and metabolic stress. Microthrombus deposition, limited perfusion, mitochondrial malfunction, and decreased energy generation all interact to maintain metabolic imbalance and cause tissue damage over time.

**Figure 3:**
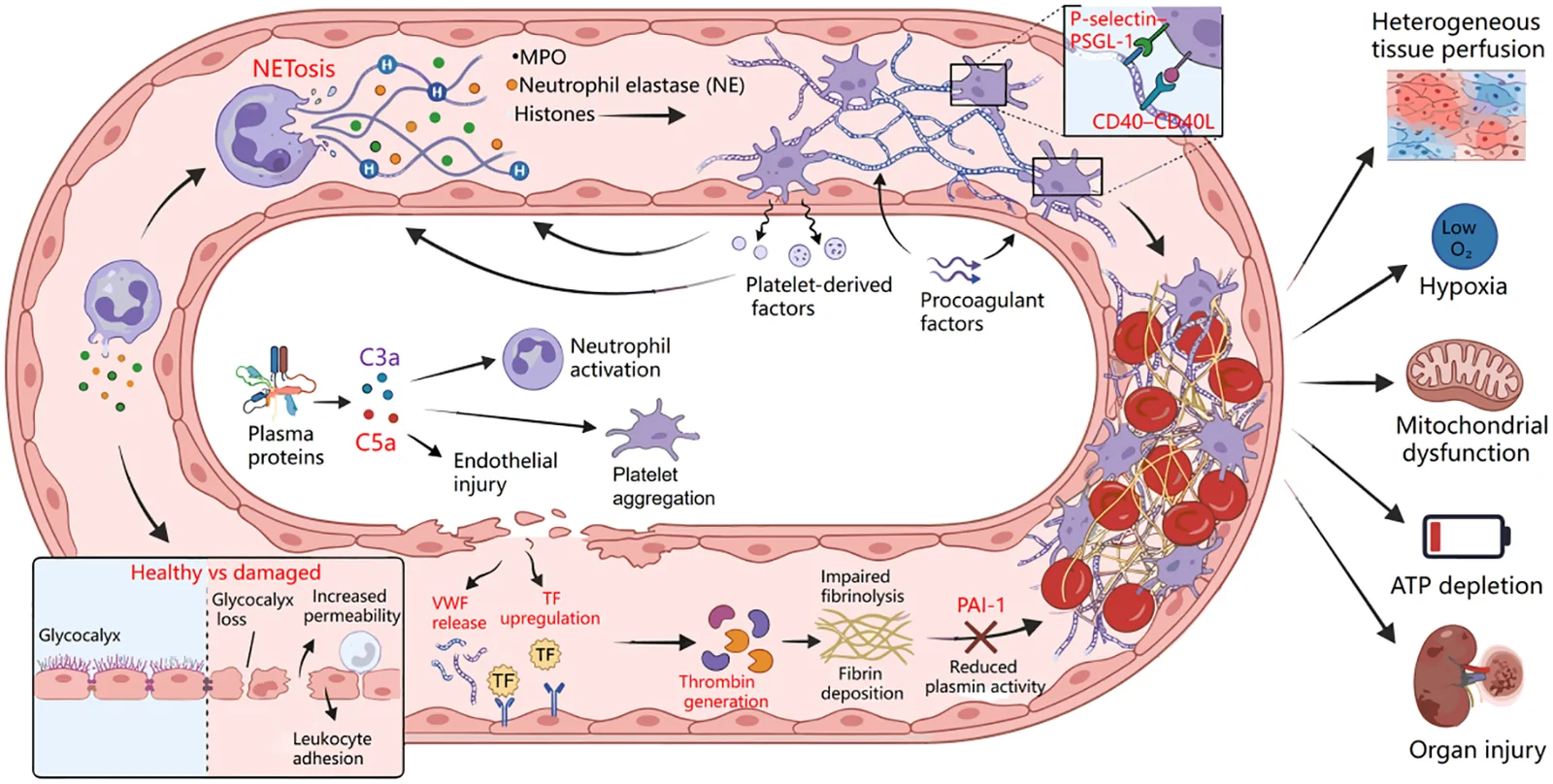
NET–platelet–complement interactions promote immunothrombosis and microvascular injury. Neutrophil NETosis releases extracellular DNA, histones, myeloperoxidase (MPO), and neutrophil elastase into the capillary lumen. NETs provide a scaffold for platelet adhesion and activation through P-selectin–PSGL-1 and CD40L–CD40 interactions. Platelet activation further supports NET formation and local coagulation. Complement activation generates C3a and C5a, which promote neutrophil activation, endothelial injury, and platelet aggregation. Injured endothelium undergoes procoagulant conversion, characterized by glycocalyx loss, increased permeability, leukocyte adhesion, von Willebrand factor (VWF) release, and tissue factor (TF) expression. Coagulation activation drives thrombin generation and fibrin deposition, while impaired fibrinolysis, mediated by increased PAI-1 and reduced plasmin activity, limits fibrin degradation. These coupled processes generate NET-, platelet-, fibrin-, and red blood cell-rich microthrombi, causing capillary obstruction, heterogeneous perfusion, tissue hypoxia, mitochondrial dysfunction, ATP depletion, and organ injury.

The effects of energy deficiency and microcirculatory dysfunction vary by organ. High mitochondrial energy output is essential for cardiomyocytes, and decreased perfusion is linked to arrhythmias and poor contractility. Acute kidney injury is linked to hypoperfusion, and renal tubular epithelial cells are extremely vulnerable to hypoxia. Perfusion instability, which is associated with neurological dysfunction, can affect the central nervous system. enhanced endotoxin translocation and enhanced systemic inflammation are linked to intestinal and liver barrier failure (100, 101). Therefore, a terminal pathway of immunothrombosis-associated damage is microvascular perfusion failure and energy-metabolism imbalance, which are linked to multi-organ dysfunction, elevated SOFA scores, and unfavorable outcomes (102) (**Figure 3**).

## 5 Organ phenotypes: variations in immune-endothelial-coagulation coupling expression

The immune-endothelial-coagulation coupling does not result in an organ-specific damage pattern. The vascular endothelium and microcirculatory structures are first revealed when systemic inflammatory signals reach the bloodstream. How the same diseased network is translated into different clinical phenotypes depends on organ-specific variations in barrier design, energy requirement, blood-flow regulation, and resident immune landscapes (21). This network primarily manifests as poor gas exchange in the lung, decreased perfusion reserve and load imbalance in the heart, tubular energy failure in the kidney, disruption of the neurovascular unit in the brain, and conversion from target-organ involvement to a secondary source of inflammatory amplification along the liver-gut axis (103). Therefore, multi-organ dysfunction should be understood as the spatial expression of a shared immune-vascular-coagulation network across many organ microenvironments rather than as a parallel accumulation of distinct problems. Importantly, similar organ-level phenotypes do not necessarily imply identical upstream mechanisms across viral pathogens. In coronavirus-associated severe pneumonia, cardiac, renal, neurological, hepatic, and gastrointestinal injury may occur in the setting of prominent systemic endothelial activation, coagulation abnormalities, and microvascular immunothrombosis (104). In severe influenza, comparable organ dysfunction may arise more strongly from extensive pulmonary epithelial injury, systemic inflammatory responses, hypoxemia, hemodynamic stress, and secondary infection, with coagulation activation acting as one component of a broader injury process (105). RSV/HMPV-associated systemic deterioration is more often linked to severe airway obstruction, respiratory failure, and hypoxic cardiopulmonary stress, whereas adenovirus may produce organ injury through extensive tissue necrosis, DAMP release, and sustained inflammatory signaling. In selected herpesvirus infections, virus-specific tissue involvement and impaired cellular immune control may contribute substantially to extrapulmonary disease, while some emerging viral infections preferentially produce endothelial leakage, shock, and hypoperfusion (106). The organ phenotypes described below should therefore be interpreted as convergent outcomes whose dominant upstream drivers vary according to pathogen biology and host context.

### 5.1 Lung: ARDS and pulmonary microthrombosis

Alveolar inflammatory exudation alone does not fully account for the severity of viral pneumonia-associated ARDS. Pulmonary microvascular immunothrombosis and endothelial dysfunction redistribute capillary perfusion, aggravating ventilation–perfusion mismatch, diffusion limitation, and physiological dead space (107, 108). Viral pneumonia-associated ARDS can therefore be viewed as a composite phenotype in which alveolar–capillary barrier failure and microvascular perfusion abnormalities act in parallel: epithelial injury contributes predominantly to protein-rich exudation and reduced lung compliance, whereas microvascular obstruction contributes to perfusion heterogeneity and persistent hypoxemia.

### 5.2 Heart: coronary microcirculatory dysfunction and myocardial injury

Elevated troponin levels, arrhythmias, and varied degrees of cardiac dysfunction are common indicators of cardiovascular involvement in viral pneumonia (109). In the majority of critically ill individuals, myocardial injury occurs in the context of ARDS, hypoxemia, and persistent systemic inflammation, which is consistent with secondary myocardial injury, even if myocarditis-like phenotypes may be seen in a subset of patients (110). Cardiac loading circumstances and myocardial oxygen supply are both affected by pulmonary pathology. Myocardial oxygen transport is restricted by prolonged hypoxemia, and the right ventricle is more susceptible to dilatation and dysfunction due to increased pulmonary vascular resistance and changes in intrathoracic pressure brought on by mechanical ventilation (111).

Myocardial perfusion reserve is decreased when coronary microvascular vasomotor control is compromised by systemic inflammation and endothelial dysfunction. The local oxygen supply–demand imbalance is made worse by immunothrombus deposition in the coronary microcirculation (112). Biochemical assessment complements this functional picture: hs-cTnI/hs-cTnT reflects cardiomyocyte injury, whereas NT-proBNP provides information on ventricular wall stress and hemodynamic load (113). In cardiomyocytes, hypoxia, inflammatory signaling, and catecholamine stress also interfere with mitochondrial oxidative phosphorylation, which lowers ATP-generating efficiency and increases calcium dyshomeostasis and electrophysiological instability (114). Therefore, it is more accurate to think of viral pneumonia-associated myocardial injury as a functional failure process caused by hypoxia burden, pulmonary circulation–right heart uncoupling, coronary microcirculatory dysfunction, and myocardial energy-metabolism imbalance.

### 5.3 Kidney: medullary hypoxic vulnerability and AKI

A typical extrapulmonary symptom of severe viral pneumonia is acute kidney damage (AKI), which is clinically defined by elevated blood creatinine, decreased urine production, and progression of kidney disease: stages of Improving Global Outcomes (KDIGO) and, in extreme situations, a greater need for continuous renal replacement therapy (CRRT) (115). Viral pneumonia-associated AKI is influenced by the combination of lung-derived inflammatory spillover, systemic hypoxemia, and renal microcirculatory dysfunction, as opposed to kidney damage caused exclusively by hypotension (116).

The renal medulla functions near a hypoxic threshold, making the kidney extremely susceptible to microcirculatory disruption. Early impairment of the medullary oxygen reserve occurs when reduced systemic oxygen delivery is caused by decreased pulmonary gas exchange. The circulation carries DAMPs, coagulation signals, and lung-derived inflammatory mediators to the glomerular and peritubular capillary endothelium, where they cause local hypoxia and heterogeneous perfusion (117). Microthrombus deposition exacerbates peritubular oxygen deprivation and further decreases effective blood flow (118).

High ATP availability is essential for solute transport and polarity maintenance in renal tubular epithelial cells. Reduced ATP production, loss of epithelial polarity, and compromised transport capacity are the outcomes of oxidative phosphorylation being restricted by hypoxia and mitochondrial malfunction (119). Urinary β2-microglobulin, kidney injury molecule-1 (KIM-1), and neutrophil gelatinase-associated lipocalin (NGAL) provide complementary evidence of tubular injury and have been linked to AKI severity (88). Therefore, it is better to think of viral pneumonia-associated AKI as a process where renal medullary hypoxic vulnerability is increased by systemic hypoxemia, microcirculatory obstruction, and tubular energy failure rather than as reduced renal perfusion alone.

### 5.4 Brain: neurovascular unit dysfunction

Clinical signs of neurological involvement in viral pneumonia include delirium, stroke-like events, fluctuating consciousness, and cognitive deterioration throughout recovery (120). Unlike conventional viral encephalitis, these symptoms typically occur in the context of persistent hypoxic burden and systemic inflammation, suggesting that the central nervous system may function more as a recipient of systemic illness than as an isolated infectious target. One crucial barrier in this process is the blood–brain barrier (BBB). Tight-junction stability is decreased, selective barrier permeability is compromised, and peripheral immune mediators are allowed to enter the brain parenchyma when inflammatory cytokines, complement signals, and hypoxia stress are coupled (121). Alterations in glial fibrillary acidic protein (GFAP), neurofilament light chain (NfL), and S100B offer complementary indicators of brain tissue damage and barrier breakdown (122).

Imaging and perfusion anomalies are the primary indicators of cerebral microcirculatory dysfunction. Regional blood flow is diverse due to microthrombus development and decreased endothelial responsiveness, with sensitive areas experiencing intermittent or focal hypoperfusion (123). Microscale perfusion disruption is correlated with diffusion-restricted lesions, microbleeds, and white matter hyperintensities on MRI (124). At this point, limited oxygen delivery and energy deficiency are more important factors controlling neuronal damage than exposure to inflammatory mediators alone (125). After that, a second wave of neuroimmune amplification can develop. Pro-inflammatory cytokine release is elevated, inflammasome signaling is maintained, and microglia transition from homeostatic surveillance to a reactive state (126). A feed-forward loop created by interactions between neurons, glial cells, and microvessels impairs synaptic function, neurotransmitter balance, and network connection, which may be the cause of delirium and long-term cognitive impairment (127).

Therefore, it is more accurate to think of viral pneumonia-related brain damage as neurovascular unit malfunction. Neuroimmune activation prolongs tissue damage, microcirculatory dysfunction limits energy delivery, and barrier failure allows inflammatory entry. Instead of a pattern dominated by direct viral invasion, this phenotype represents the convergence of vascular and immunological systems.

### 5.5 Liver–gut axis: dual roles of hepatic filtering and intestinal amplification

Increased ALT and AST, abnormalities in ALP, GGT, and total bilirubin, hypoalbuminemia, extended prothrombin time, and gastrointestinal dysfunction are all indicators of liver-gut axis involvement in viral pneumonia (128). These alterations are more consistent with the combined effects of lung-derived inflammatory spillover, hypoxic load, hepatic sinusoidal microcirculatory dysfunction, and intestinal barrier disruption than with isolated viral hepatitis or primary gastrointestinal infection (129).

Signals relating to coagulation and inflammation are filtered through the liver. Intrahepatic microcirculatory perfusion and liver sinusoidal endothelial cell (LSEC) activity are disturbed when lung-derived inflammatory mediators, DAMPs, and coagulation-related signals enter the bloodstream (130). Hepatocellular metabolic stress, decreased bile acid transport, and local ischemia may all be made worse by immunothrombosis (131). Biochemical patterns help distinguish different components of hepatic involvement. ALT and AST primarily reflect hepatocellular injury, whereas abnormalities in ALP, GGT, and total bilirubin are more consistent with cholestatic dysfunction or impaired bile excretion. Prolonged PT/INR and reduced albumin provide additional evidence of impaired hepatic synthetic function (132).

Systemic inflammation may be amplified by the gut. Tight-junction structures like ZO-1, occludin, and claudins are disrupted by hypoperfusion, inflammatory cytokines, and hypoxic stress, allowing gut-derived lipopolysaccharide (LPS) and microbial-associated molecular patterns to enter the portal and systemic circulation (133). I-FABP, D-lactate, and circulating LPS provide complementary indicators of intestinal epithelial injury, increased barrier permeability, and endotoxin translocation (134). Following transport to the liver, LPS triggers the release of TNF-α, IL-6, IL-1β, and other inflammatory mediators by activating Kupffer cells and circulating immune cells via TLR4–NF-κB signaling. Thus, through barrier disruption, endotoxin translocation, and Kupffer cell activation, the liver-gut axis is not only a target of systemic injury but also a feedback amplifier of inflammation (135).

### 5.6 Clinical applicability of biomarkers: established, context-dependent, and investigational markers

The biomarkers discussed throughout this Review differ substantially in their level of clinical implementation and should not be interpreted as having equivalent diagnostic or prognostic maturity. Routinely available laboratory and physiological measures, including complete blood cell counts, C-reactive protein, standard coagulation parameters such as D-dimer and prothrombin time/international normalized ratio, serum lactate, serum creatinine and urine output, cardiac troponin, natriuretic peptides, and routine liver-function tests, are already incorporated into clinical assessment according to the presenting syndrome and suspected organ involvement. Some additional biomarkers, including procalcitonin, ferritin, IL-6, von Willebrand factor, NGAL, and selected neurological injury markers, may be clinically available but their use is context dependent and they should generally complement, rather than replace, conventional clinical and laboratory assessment (136). In contrast, biomarkers that more directly reflect the proposed immune–endothelial–coagulation mechanisms remain predominantly investigational in severe viral pneumonia. These include endothelial and glycocalyx-associated markers such as angiopoietin-2, syndecan-1, soluble thrombomodulin, heparan sulfate, and hyaluronan; NET-associated markers such as circulating cell-free DNA, MPO–DNA complexes, and citrullinated histone H3; DAMP-related markers such as mitochondrial DNA, HMGB1, and S100A8/A9; complement activation fragments such as C3a and C5a; and other mechanistic biomarkers including PAI-1, KIM-1, I-FABP, D-lactate, and circulating LPS (137). At present, these markers are most useful for mechanistic research, phenotype discovery, and biomarker-enriched clinical studies, because standardized assays, validated thresholds, longitudinal reproducibility, and evidence that biomarker-guided treatment improves outcomes remain limited. Accordingly, biomarker interpretation in severe viral pneumonia should distinguish routine markers of disease severity and organ dysfunction from emerging markers intended to characterize the underlying pathological phenotype (**Table 2**).

**Table 2:** Clinical implementation status of biomarkers discussed in severe viral pneumonia.

Category	Biomarkers	Main clinical/research role	Current status
Inflammation	CBC/neutrophil/lymphocyte counts, CRP	Inflammatory burden, severity assessment	Routine clinical use
Inflammation/infection	Procalcitonin	Adjunct to infection/antimicrobial assessment	Clinical, context-dependent
Inflammation	Ferritin, LDH, IL-6	Hyperinflammation/severity characterization	Clinical availability varies; mainly adjunctive
Coagulation	D-dimer, PT/INR, platelet count	Coagulation abnormalities/thrombotic risk	Routine clinical use
Perfusion	Lactate	Tissue hypoperfusion/severity/resuscitation	Routine clinical use
Cardiac	hs-cTnI/hs-cTnT, NT-proBNP	Myocardial injury/hemodynamic stress	Clinical use when indicated
Renal	Serum creatinine, urine output	AKI diagnosis/staging	Routine/established clinical use
Renal	NGAL	Early tubular injury/risk assessment	Emerging/context-dependent (138)
Renal	KIM-1, urinary β2-microglobulin	Tubular injury/mechanistic phenotyping	Predominantly investigational (139)
Hepatic	ALT, AST, bilirubin, albumin, PT/INR	Hepatic injury/function	Routine clinical use
Endothelial/glycocalyx	Ang-2, syndecan-1, sTM, heparan sulfate, hyaluronan	Endothelial activation/glycocalyx injury	Predominantly investigational (140)
NETs	cfDNA, MPO–DNA, Cit-H3	NET formation/immunothrombotic phenotype	Investigational (141)
DAMPs	mtDNA, HMGB1, S100A8/A9	Cell injury/innate immune activation	Investigational (142)
Complement	C3a, C5a	Complement activation phenotype	Investigational in this context (143)
Fibrinolysis	PAI-1	Fibrinolytic suppression	Predominantly investigational (144)
Neurological	GFAP, NfL, S100B	Neural/glial injury	Context-dependent/emerging (145)
Intestinal barrier	I-FABP, D-lactate, LPS	Gut barrier injury/translocation	Predominantly investigational (146)

## 6 Stratified intervention strategies targeting the coupled network

Within the immune–endothelial–coagulation framework, treatment of severe viral pneumonia may be conceptualized as a stage- and phenotype-informed combination of pathogen-directed therapy, host-response modulation, vascular–coagulation management, and organ support rather than as a fixed combination of interventions. During earlier disease stages, viral replication, epithelial injury, and local inflammatory triggers may have greater relative importance, whereas inflammatory spillover, endothelial activation, procoagulant conversion, and immunothrombotic processes may become increasingly relevant as severe systemic disease develops (21). This mechanistic framework may therefore help generate hypotheses regarding therapeutic timing and patient stratification; however, prospective studies are required to determine whether targeting specific nodes of the coupled network improves clinical outcomes.

### 6.1 Stage-adapted therapy: from antiviral treatment to host-response modulation

Antiviral therapy’s therapeutic benefit is concentrated in the early stages, when viral replication is most prevalent (147). Reduced viral burden can attenuate the start of immune–endothelial–coagulation coupling by limiting virus-associated cellular damage, DAMP release, and downstream inflammatory cytokine production. Oseltamivir, peramivir, baloxavir, and similar medications are mostly utilized during active viral replication in influenza virus infection. Remdesivir and nirmatrelvir/ritonavir both rely on an early treatment window to be effective in treating SARS-CoV-2 infection (148). These medicines help reduce the trigger burden for eventual host-response dysregulation in addition to controlling pathogens.

Therapeutic priorities change from pathogen control to host-response modulation once the replication-dominant period has passed (149). Viral suppression is typically insufficient to stop the evolution of the disease at this point because systemic inflammation, endothelial damage, and coagulation activation form a mutually reinforcing pathological network (150). Glucocorticoids, such as dexamethasone, attenuate inflammatory spillover and tissue damage, decrease cytokine release and immune-cell recruitment, and regulate NF-κB-associated inflammatory signaling (151). For patients with hyperinflammatory phenotypes, JAK inhibitors like baricitinib and IL-6 receptor antagonists like tocilizumab and sarilumab are important host-response-modulating treatments (152). For patients with hyperinflammatory features, the selection and timing of host-response-modulating therapies may be informed by the overall clinical context, including inflammatory markers, oxygenation status, lymphocyte counts, secondary infection risk, and disease stage, while recognizing that validated biomarker-defined treatment thresholds remain limited (153).

### 6.2 Targeting the endothelial–coagulation axis: anticoagulation, microcirculatory protection, and restoration of endothelial homeostasis

When endothelial injury and procoagulant conversion become prominent features of severe disease, preservation of microcirculatory function and vascular homeostasis may represent relevant therapeutic considerations (154). Immunothrombotic processes are associated with tissue hypoxia, perfusion abnormalities, and microvascular injury (155). Anticoagulation in appropriately selected patients and strategies aimed at preserving endothelial function may therefore form part of a phenotype-informed therapeutic approach; however, their indications, timing, and net clinical benefit require individualized assessment and further prospective validation.

Widely used anticoagulant techniques include low-molecular-weight heparin and unfractionated heparin, which may lessen the risk of thrombotic problems, decrease microthrombus production, and attenuate excessive coagulation cascade activation (156). Potential benefit may be greater in appropriately selected patients with hypercoagulability, elevated D-dimer, or increased thrombotic risk, although these features alone do not establish treatment benefit (157). Anticoagulation should not be used as a fixed module for all critically ill patients; instead, it should be individualized according to coagulation phenotype and risk stratification (158).

Endothelial protection targets upstream processes in immunothrombosis (159). Preservation of glycocalyx integrity, attenuation of endothelial inflammatory activation, stabilization of vascular permeability, and restoration of the anticoagulant phenotype may limit leukocyte adhesion, platelet aggregation, and local coagulation initiation (160). Complement inhibition, kallikrein–kinin system modulation, oxidative-stress-targeted intervention, and glycocalyx protection may represent candidate strategies for selected patient subtypes (161). Accordingly, anticoagulation and candidate endothelial-protective strategies may be conceptualized within the broader continuum of inflammatory spillover, endothelial injury, and immunothrombosis, while the clinical effectiveness of endothelial-directed interventions remains to be established.

### 6.3 NETs, complement, NLRP3, and precision stratification in host-directed therapy

One possible treatment approach for severe viral pneumonia is host-directed therapy that targets important immune-endothelial-coagulation coupling nodes. NETs, complement pathways, the NLRP3 inflammasome, and mitochondrial injury-associated signals lie at the intersection of inflammatory amplification, endothelial injury, and immunothrombosis, making them mechanistically plausible candidate therapeutic targets (150). NET-directed approaches, including DNase and PAD4 inhibition, are designed to reduce DNA scaffold formation, histone-mediated cytotoxicity, and platelet adhesion, thereby attenuating immunothrombosis and endothelial injury (162). Complement inhibition, including C5 blockade with eculizumab or ravulizumab and C3-targeted approaches, may limit C3a/C5a-mediated neutrophil recruitment, endothelial activation, and platelet aggregation (163). IL-1 pathway blockade, including anakinra, and NLRP3 inhibition may reduce IL-1β and IL-18 release, pyroptosis, and sustained DAMP liberation (164). Metabolic-protective strategies targeting mitochondrial injury and mtDNA release may attenuate cGAS–STING-associated inflammatory signaling and oxidative stress, thereby improving tissue tolerance to hypoxic and inflammatory stress (165).

**Figure 4:**
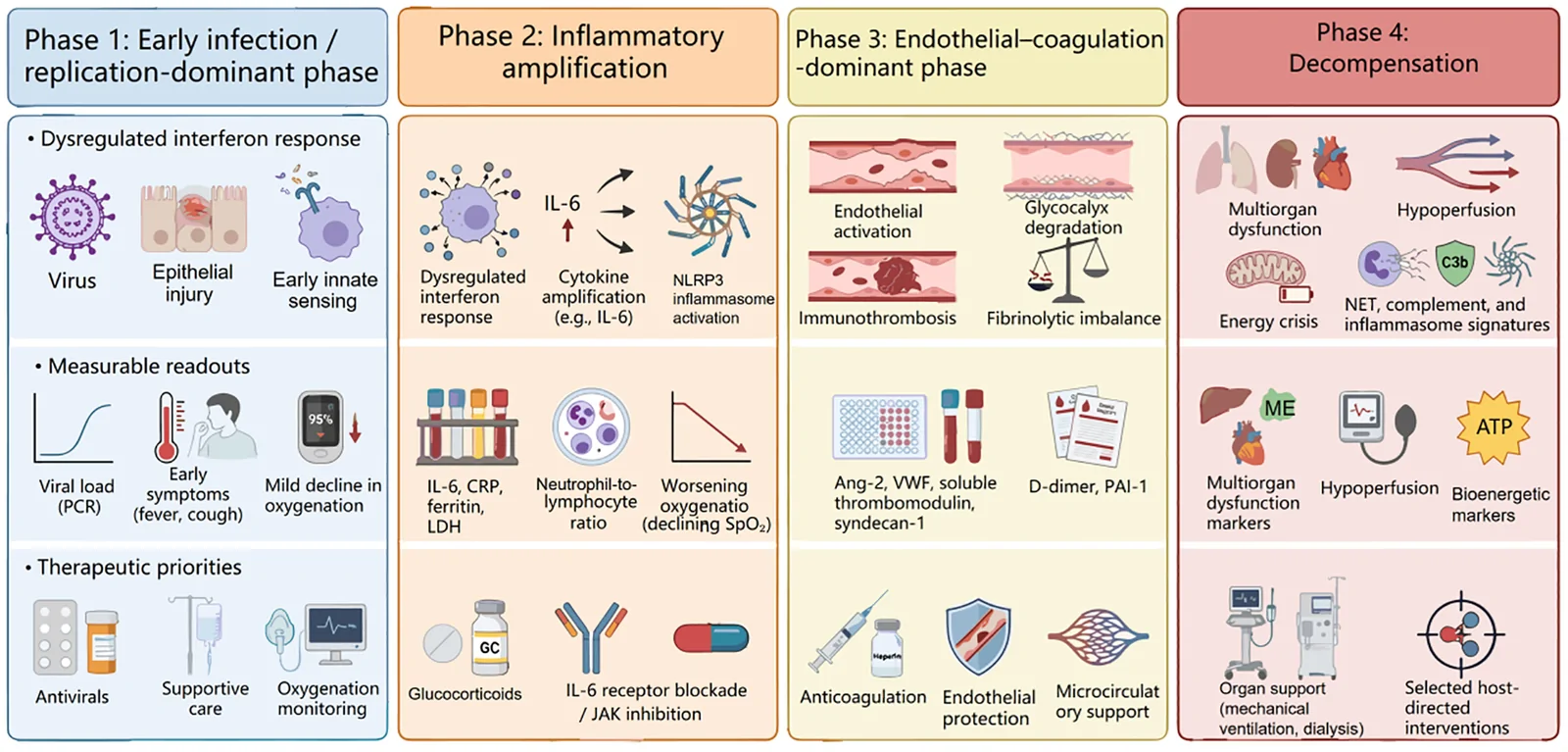
Phenotype-guided intervention across disease stages in severe viral pneumonia. This framework summarizes stage-adapted intervention according to dominant pathological drivers, measurable readouts, and therapeutic priorities in severe viral pneumonia. During the early infection or replication-dominant phase, viral replication, epithelial injury, and early innate sensing predominate; relevant readouts include viral load, early symptoms, and mild oxygenation decline, supporting antiviral therapy, supportive care, and oxygen monitoring. During the inflammatory amplification phase, dysregulated interferon responses, cytokine amplification, and NLRP3 activation are reflected by IL-6, CRP, ferritin, LDH, neutrophil-to-lymphocyte ratio, and worsening oxygenation, supporting glucocorticoids, IL-6 receptor blockade, and JAK inhibition. In the endothelial–coagulation-dominant phase, endothelial activation, glycocalyx degradation, immunothrombosis, and fibrinolytic imbalance are assessed by Ang-2, VWF, soluble thrombomodulin, syndecan-1, D-dimer, and PAI-1, supporting anticoagulation, endothelial protection, and microcirculatory support. In the decompensation phase, multi-organ dysfunction, hypoperfusion, energy crisis, NET/complement signatures, and inflammasome activity guide organ support and selected host-directed interventions.

Most of these strategies remain in mechanistic or early translational stages, with uncertain target populations, therapeutic windows, safety boundaries, and combination regimens (166). Whether durable clinical benefit can be obtained through single-node intervention within a complex coupled network remains to be established in prospective studies and mechanism-embedded clinical trials (167). Host-directed therapy should therefore be viewed as a pathology-stratified precision intervention strategy rather than a broadly applicable standard treatment at present. Its translational value will depend on the identification of NET-dominant, complement-activated, inflammasome-driven, or endothelial injury-dominant phenotypes and on stratified interruption of the immune–endothelial–coagulation network (**Figure 4**).

## 7 Research challenges and future directions

The immune–endothelial–coagulation coupling framework has been used to explain how viral pneumonia progresses from localized infection to multi-organ dysfunction, but the current evidence base remains dominated by associative observations (168). In addition, the evidence supporting individual components of this framework is uneven across viral pathogens, with endothelial injury, NET-associated immunothrombosis, and coagulation dysregulation being particularly well characterized in SARS-CoV-2 infection, whereas their relative importance in influenza, RSV, adenovirus, and other viral pneumonias requires further pathogen-specific validation (169). Inflammatory amplification, endothelial injury, complement activation, NET release, and coagulation abnormalities have been documented; however, their causal ordering and dominant roles across disease stages and patient subtypes remain insufficiently defined (170). In order to assist mechanism-based clinical stratification and therapeutic decision-making, future research should therefore determine whether these associations can be resolved into reproducible temporal and causal relationships across the course of disease, thereby providing a stronger basis for mechanism-based stratification and therapeutic testing. Direct comparative studies across viral pathogens will also be required to determine whether similar organ phenotypes arise from shared downstream pathways or from pathogen-specific upstream mechanisms with different relative contributions of epithelial injury, inflammation, endothelial dysfunction, coagulation activation, and hypoperfusion.

### 7.1 Dynamic mechanisms and stratification systems

Multi-organ damage linked to viral pneumonia develops as an ongoing pathological process, but it is still unclear what the turning moments are that cause the disease to worsen. Increased inflammatory mediators, coagulation problems, endothelial damage, and organ dysfunction are frequently found simultaneously in single-time-point or cross-sectional investigations, making temporal ordering challenging to establish and causal inference poor. Time-series study designs are therefore required to resolve these limitations. Longitudinal cohorts with multi-time-point sampling of blood, respiratory, and tissue compartments may help reconstruct disease trajectories. Single-cell and spatial transcriptomic analyses delineate spatiotemporal reprogramming of immune, endothelial, and stromal cells, whereas plasma proteomics, metabolomics, and coagulation profiling capture dynamic remodeling of inflammation–metabolism–coagulation networks (171). Trajectory modeling, dynamic network analysis, and causal mediation analysis have been used to identify key nodes in the inflammatory spillover–endothelial injury–immunothrombosis–organ dysfunction axis and to define candidate therapeutic windows (166).

Mechanism-based stratification provides a bridge between causal-chain reconstruction and clinical decision-making. Current clinical assessment systems use oxygenation indices, inflammatory markers, and coagulation parameters to quantify disease severity, but these variables do not adequately distinguish distinct pathological drivers. Multidimensional biomarker integration has therefore been proposed to establish mechanism-based stratification systems (172). Inflammatory cytokine profiles, endothelial injury markers such as Ang-2 and sTM, complement activation fragments, NET-associated molecules including cfDNA, MPO–DNA, and Cit-H3, coagulation–fibrinolysis indices, circulating DAMPs, and metabolomic signatures can be assembled into composite biomarker panels. However, most endothelial-, NET-, complement-, DAMP-, and multi-omics-based markers remain investigational and have not yet achieved the assay standardization, validated clinical thresholds, external validation, or prospective evidence required for routine phenotype-guided treatment (173). These markers should therefore be distinguished from conventional laboratory indices, such as blood cell counts, CRP, D-dimer, lactate, serum creatinine, cardiac troponin, and routine hepatic and coagulation tests, which are already used clinically to assess disease severity and organ dysfunction. Unsupervised clustering, latent class analysis, and machine-learning models may then be used to explore or identify candidate hyperinflammatory, endothelial injury-dominant, immunothrombotic, and hypoperfusion–metabolic imbalance phenotypes (174). The reliability of these stratification models depends on external validation, dynamic threshold definition, replication in independent cohorts, and time-series data analysis. Biomarker research is consequently moving toward evaluating whether mechanistic profiles can support therapeutic matching, in which specific mechanistic phenotypes are linked to corresponding intervention strategies to support stratified treatment decisions (175).

### 7.2 Window-based intervention and translational research

After candidate mechanistic drivers have been identified, translational research could examine whether interventions are more effective when applied within biologically defined therapeutic windows. Current treatment strategies are largely stratified by clinical severity, whereas therapeutic response may also depend on the underlying pathological phase (176). Within the conceptual framework proposed here, antiviral therapy is most closely aligned with the viral-replication phase, immunomodulation with inflammatory amplification, anticoagulation or endothelial-directed approaches with selected immunothrombotic phenotypes, and organ support with physiological decompensation. These proposed alignments should be regarded as testable hypotheses rather than established treatment algorithms and require prospective validation. Mechanism-guided trial designs may help determine whether such stage- and phenotype-based strategies improve treatment precision. Platform trials and adaptive randomized trial frameworks provide potential approaches for comparing multiple interventions within a shared trial infrastructure. Mechanism-embedded trials may additionally incorporate longitudinal assessment of inflammatory, endothelial, coagulation, and NET-related markers to determine whether candidate target pathways are modified during treatment. Enrichment designs could be used to investigate whether patient selection according to mechanistic phenotypes improves the ability to identify treatment-responsive subgroups. Future endpoint frameworks may also benefit from incorporating mechanism-relevant measures, including microcirculatory function, organ-function trajectories, coagulation–fibrinolysis recovery, inflammatory-resolution kinetics, and long-term functional outcomes, in addition to conventional clinical endpoints (177). Integrating temporal profiling, mechanistic stratification, and prospective trial designs may ultimately clarify whether mechanism-guided precision intervention improves outcomes in severe viral pneumonia.

## 8 Conclusion

Severe viral pneumonia should be viewed as a systemic immunovascular pathological process that is triggered by pulmonary infection, fueled by dysregulated host immunity, and exacerbated by endothelial damage and coagulation activation rather than as an infectious disease limited to the respiratory system (178). Local pulmonary inflammation is transformed into systemic microvascular injury by a combination of dysregulated antiviral responses, alveolar–capillary barrier failure, DAMP/NET release, endothelial glycocalyx degradation, platelet–complement interactions, and immunothrombosis. Tissue hypoxia, disruption of energy metabolism, and organ decompensation are subsequently imposed by microthrombus deposition and microcirculatory perfusion failure, creating a terminal pathway towards multi-organ dysfunction (179). The principal conceptual contribution of this Review is therefore not the identification of a previously unrecognized pathway, but the integration of established immune, endothelial, coagulation, microcirculatory, and organ-specific mechanisms into a temporally ordered framework that connects pulmonary disease initiation with systemic progression and mechanism-guided intervention.

This framework may provide a conceptual basis for investigating stage-adapted and phenotype-informed management strategies. Antiviral therapy, host-response modulation, anticoagulation in appropriately selected patients, candidate endothelial-protective approaches, and organ support may have complementary roles at different stages of illness; however, their use should remain grounded in established clinical indications and available evidence rather than in the mechanistic framework alone. The clinical value of aligning interventions with dominant pathological mechanisms and patient phenotypes remains an important hypothesis for prospective evaluation. Future studies should determine whether immune–endothelial–coagulation coupling can be translated from an integrative mechanistic model into a clinically actionable framework (180). Longitudinal cohorts, multi-omics integration, externally validated stratification biomarkers, and mechanism-embedded clinical trials may help clarify causal relationships among inflammatory spillover, endothelial injury, and immunothrombosis and evaluate proposed therapeutic windows. With further validation, this framework may contribute to the development of more mechanism-informed approaches to severe viral pneumonia.
